# Common and disorder-specific upregulation of the inflammatory markers TRAIL and CCL20 in depression and schizophrenia

**DOI:** 10.1038/s41598-021-98769-0

**Published:** 2021-09-28

**Authors:** Federica Klaus, Karoline Guetter, Rebecca Schlegel, Tobias R. Spiller, Erich Seifritz, Flurin Cathomas, Stefan Kaiser

**Affiliations:** 1 Department of Psychiatry, Psychotherapy and Psychosomatics, Psychiatric Hospital, University of Zurich, Lenggstrasse 31, 8032 Zürich, Switzerland; 2 Department of Psychiatry, University of California San Diego, San Diego, USA; 3 Department of Consultation-Liaison Psychiatry and Psychosomatic Medicine, University Hospital Zurich, University of Zurich, 8091 Zurich, Switzerland; 4 Fishberg Department of Neuroscience, Friedman Brain Institute, Icahn School of Medicine at Mount Sinai, New York, USA; 5 Division of Adult Psychiatry, Department of Psychiatry, Geneva University Hospitals, Chemin du Petit-Bel-Air, 1225 Chêne-Bourg, Switzerland

**Keywords:** Cytokines, Immunology, Biomarkers, Signs and symptoms

## Abstract

Schizophrenia (SZ) and major depressive disorder (MDD) are severe mental disorders, which have been associated with alterations of the peripheral inflammatory network. However, studies for both disorders have not been fully consistent and have focused on few canonical markers with high relevance to the innate immune system, while the role of the adaptive immune system is studied less. Furthermore, it is unclear to what extent inflammatory abnormalities are diagnosis-specific or transdiagnostic. The purpose of this study was to investigate 75 peripheral inflammatory markers including the acute phase protein high-sensitivity C-reactive protein (hsCRP) in patients with MDD (n = 37), SZ (n = 42) and healthy controls (HC) (n = 17), while considering possible confounders and correcting rigorously for multiple testing in group comparisons. We identified C–C chemokine ligand 20 (CCL20) and tumor necrosis factor-related apoptosis-inducing ligand (TRAIL) as the inflammatory markers with significant group differences after controlling for multiple comparisons and adjusting for BMI, sex and smoking as confounders. TRAIL was elevated in both MDD and SZ compared to HC. CCL20 was specifically increased in SZ compared to MDD and HC. There were no significant group differences in hsCRP after correcting for multiple testing. Finally, we observed no significant correlations among CCL20, TRAIL and CRP. TRAIL is a transdiagnostic marker for SZ and MDD, with both markers being independent from CRP and body mass index (BMI). CCL20 may be a novel and specific biomarker of schizophrenia, but an influence of antipsychotic medication cannot be excluded. Identifying novel markers in mental disease bears the potential for future research towards novel treatment strategies by modifying inflammation-related processes.

## Introduction

Schizophrenia (SZ) and major depressive disorder (MDD) are severe chronic mental illnesses with high socioeconomic and individual burden. Treatment options are not satisfying^1^, mainly due to the not yet elucidated pathophysiological mechanisms.

The inflammatory network in the periphery and brain has been associated with MDD^2–8^ and SZ^9–24^. Increased peripheral levels of the pro-inflammatory cytokines TNF alpha (TNF-α) and Interleukin 6 (IL-6) as well as the acute-phase protein C-reactive protein (CRP) have repeatedly been reported^24–27^. There are inconsistencies across individual studies, with conflicting findings regarding differences of individual markers between diagnoses. This is partly a consequence of the inherent heterogeneity of the disorders themselves (e.g. the illness stage), but also due to the many disease-independent confounders such as body weight, possible comorbidities and effects of medication. However, a recent large meta-analysis revealed a consistent elevation of inflammatory markers in MDD, among them CRP, IL-3, IL-6, IL-12, IL-18, sIL-2R and TNF-α^28^ and a study assessing 43 meta-analyses reported increased levels of inflammatory markers, including CRP, IL-6, IL12 in both MDD and SZ^26^. Differences between acute versus chronic and exacerbated versus remitted disease states have to be taken into account. Concerning schizophrenia, a meta-analysis reported increased levels of IFN-γ, IL-1RA, IL-1β, IL-6, IL-8, IL-12, sIL-2R, TGF-β and TNF-α in acutely ill patients (i.e. acutely psychotic) with chronic schizophrenia and a significant decrease in IL-1β, IL-4 and IL-6 following treatment^25^. In MDD, the same meta-analysis revealed increased levels of IL-6 in chronically ill patients, and a decrease of IL-6, IL-10 and IL-12 levels following treatment of acute depression^25^.

Another consideration is that there is a bias in the literature towards a focus on a few canonical cytokines and chemokines with high relevance to the innate immune system, while the role of the adaptive immune system in mental health, specifically schizophrenia and depression, is studied less^29–31^. There is evidence suggesting autoimmune components in SZ, specifically altered lymphocyte count ratios, lymphocyte infiltration in brain regions relevant to psychosis and a recent paradigm of neuronal surface antibody-mediated central nervous system disease provides an antigen-specific model linking adaptive autoimmunity to psychopathology^32^.

CRP is one of the most frequently assessed markers, which is also routinely measured in clinical practice. A recent meta-analysis on peripheral CRP in MDD identified a weak relationship between CRP and depression, depending on the quality (i.e. consideration of covariates and sample collection procedures) of studies^33^, outlining the complexity of investigating single immune parameters^34^. Compared to HC, a recent meta-analyses identified increased levels of markers for general inflammation, i.e. CRP in SZ^25,26^. CRP is an acute phase protein produced by the liver in response to cytokines of the innate immune system, among them IL-6 and TNFα^35^. CRP is a widely used marker for the general assessment of systemic inflammation, however it is confounded by several factors, including age, Body Mass Index (BMI) and smoking^36^. The broad involvement and lack of specificity of CRP itself and its modifying factors, point towards the need for new, more robust and specific inflammatory markers.

The identification of more specific inflammatory markers can be achieved by directly comparing related but separate diagnostic groups. This allows to disentangle transdiagnostic versus disorder-specific individual inflammatory markers. However, until now, to our knowledge no study has simultaneously investigated the interrelationship of a broad range of peripheral inflammation-associated cytokines and chemokines with CRP in patients with MDD and SZ.

In this study, we therefore investigated group differences between patients with MDD, SZ and healthy controls (HC) in 75 peripheral inflammatory markers including the acute phase protein CRP. The SZ patient group consisted of clinically stable patients with low positive symptoms. We hypothesized to find increased inflammatory markers in participants with SZ and MDD compared to healthy control participants, among them canonical markers, e.g. IL-6, as well as CRP, and to detect novel markers without prior reports in the literature. We specifically included a comparison of MDD vs SZ to assess specificity to these diseases. In our analysis, we considered possible confounders and corrected rigorously for multiple testing in group comparisons. We then followed up on the emerged marker’s (Tumor necrosis factor-related apoptosis-inducing ligand (TRAIL) and C–C chemokine ligand 20 (CCL20)) potential relationship with CRP as a marker for general, unspecific inflammation to evaluate their potential dependence on inflammatory processes.

## Results

### Demographics

Demographic data of the sample are shown in Table 1. Compared to HC, SZ participants showed lower levels of formal education (p = 0.048) and of personal and social functioning (p < 0.001), while MDD participants showed lower levels of personal and social performance (p < 0.001).

**Table 1:** Sociodemographic data.

	Control group	Patient group	Patient group	Main effect		Pairwise comparisons		
	(HC) (n = 17)	(SZ) (n = 42)	(MDD) (n = 37)	Test statistics		HC vs SZ	HC vs MDD	SZ vs MDD
				*F/χ*^2^*/U*	*p*	*p*	*p*	*p*
Age (years)	33.12 (9.72)	33.83 (10.26)	35.35 (11.22)	*F* = 0.331	0.719	–	–	–
Sex (male/female)	8/9	29/13	17/20	*χ*^2^ = 4.975	0.083	0.114	0.939	0.38
Ethnicity (Caucasian/African/Asian/Hispanic)	17/0/0/0	35/2/2/3	32/2/3/0	*χ*^2^ = 10.872	0.54	–	–	–
Formal education (years) ^1^	14.5 (2.28)	12.35 (3.93)	14.41 (3.29)	*F* = 4.344	**0.016**	**0.048**	0.926	**0.028**
BMI	23.56 (4.49)	26.13 (4.46)	22.75 (4.3)	*F* = 6.128	**0.003**	0.068	0.532	**0.003**
Smoking (py)	2.53 (4.71)	7.26 (8.44)	3.84 (8.76)	*F* = 2.831	0.064	–	–	–
Personal and social performance score (PSP)	96.94 (4.97)	52.64 (14.13)	58.19 (17.25)	*F* = 60.075	** < 0.001**	** < 0.001**	** < 0.001**	0.091
Patient status (oupatient/inpatient) ^2^	–	22/20	16/21	*χ2* = 0.658	–	–	–	0.417
Number of psychotic episodes ^2^	–	5.4 (5.31)	0 (0)	*U* = 18.5	–	–	–	** < 0.001**
Number of depressive episodes ^2^	–	0.07 (0.26)	3.46 (3.1)	*U* = 18.00	–	–	–	** < 0.001**
Chlorpromazine equivalents (mg/day) ^2^	–	513.33 (455.81)	2.73 (12.21)	*U* = 61.1	–	–	–	** < 0.001**
Imipramine equivalents (mg/day) ^2,3^	–	11.35 (41.81)	109.79 (95.48)	*U* = 35.6	–	–	–	** < 0.001**
Illness duration (years) ^2^	–	9.66 (8.09)	6.65 (7.43)	*U* = 560	–	–	–	**0.033**

Data are presented as means and standard deviations. Potential group differences between all three groups were investigated using ANOVA. P values lower than 0.05 are in bold, correction for multiple testing applied using FDR for pairwise comparisons. For comparison between the two patient groups, Mann Whitney U was used.

*BMI* body mass index, *HC* healthy control participants, *Py* pack years, *MDD* major depressive disorder, *SZ* Schizophrenia.

^1^Compulsory education in Switzerland is 9 years.

^2^Test statistics are only applicable to patient group.

^3^Calculation based on equivalent doses according to^72^.

In comparison to MDD, SZ participants showed fewer years of formal education (p = 0.028), a higher BMI (p = 0.003), more psychotic episodes (p < 0.001), fewer depressive episodes (p < 0.001), higher chlorpromazine equivalents (p < 0.001), and a longer illness duration (p = 0.033). The MDD and SZ groups had similar numbers of outpatients versus inpatients. No significant main effects between groups were observed in age, smoking, ethnicity and sex.

### CCL20 and TRAIL differ between diagnostic groups

**Figure 1:**
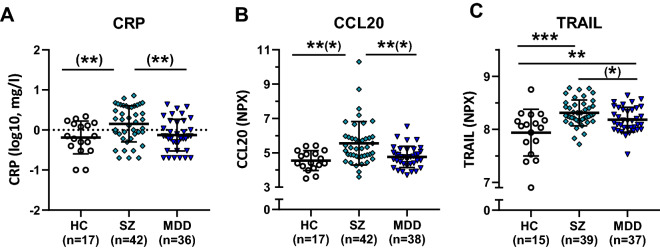
Group comparison of inflammatory markers. (**A**) CRP (log), (**B**) CCL20, (**C**) TRAIL. Units are mg/l for log10 (CRP), otherwise normalized protein expression (NPX) (an arbitrary unit on a log2-scale where a high value corresponds to a higher protein expression). *HC* healthy control, *MDD* major depression disorder, *SZ* schizophrenia, *CRP* C-reactive protein, *CCL* C–C chemokine ligand, *TRAIL* TNF (tumor necrosis factor)-related apoptosis-inducing ligand. Mean and standard deviation (SD) are depicted. After post-hoc comparison (FDR): *p ≤ 0.05, **p ≤ 0.01, ***p ≤ 0.001; asterisks in brackets indicate changes in significance after introducing BMI, sex and smoking as covariate.

We observed a significant group difference in CRP (log_10_) (F(2,92) = 6.041, p < 0.003), with post-hoc comparison revealing a significant increase in patients with SZ compared to HC (p = 0.009, Cohen’s d = 0.793) and an increase in patients with SZ compared to patients with MDD (p = 0.009, d = 0.665) with no significant difference between HC and MDD (p = 0.636, d = 0.147). To further investigate the underlying mechanisms that might drive an overall inflammatory activity represented by CRP, we analyzed group differences of inflammatory markers. Only group differences that remained significant after adjusting for multiple testing are hereafter reported (for a list of abbreviations see Supplementary Table S1 and for a full list of group comparisons of inflammatory markers see Supplementary Table S2). CCL20 (F(2,93) = 9.887, p < 0.0001) demonstrated a significant increase in patients with SZ compared to HC (p = 0.001, d = 1.024) and an increase in patients with SZ compared to MDD (p = 0.001, d = 0.796) with no difference between HC and patients with MDD (p = 0.447, d = 0.358). TRAIL (F(2,93) = 1.433, p < 0.0001) demonstrated a significant increase in patients with SZ compared to HC (p < 0.0001, d = 1.046) and an increase in patients with MDD compared to HC (p = 0.006, d = 0.692) and a significant increase in patients with SZ vs MDD (p = 0.049, d = 0.536) (Fig. 1).

We introduced smoking and sex as covariates in addition to BMI, based on their reported influence on inflammatory markers^36^. After introducing smoking, sex and BMI, the main effect of CRP (log_10_) did not remain significant (F(2,98) = 2.167, p = 0.120), while it did not alter the main results of CCL20 (F(2,90) = 6.559, p = 0.001, post hoc: HC < SZ p = 0.002, MDD < SZ p = 0.002, HC vs MDD p = 0.483) and TRAIL (F(2,90) = 6.377 , p = 0.003, post hoc: HC < SZ, p < 0.001, HC < MDD p = 0.003, SZ vs MDD p = 0.625 (new: non-significant)).

We did not include antipsychotic and antidepressant medication as a covariate, because healthy controls were medication free. Partial correlations for CCL20 and antipsychotic equivalents in the SZ group (SZ: r(s) = 0.202, p = 0.218; MDD: (s) =  − 0.432, p = 0.011) and for TRAIL and antidepressant equivalents (SZ: r(s) = 0.004, p = 0.979; MDD: (s) = − 0.090, p = 0.612) were not significant.

### BMI correlates with CRP but not with CCL20 or TRAIL

In order to investigate the relationship with the common, inflammation-dependent confounders, BMI and smoking, we correlated in a next step the two markers for which significant differences in group analysis were observed (CCL20 and TRAIL) with CRP. We performed the analysis in the three diagnostic groups separately, in order to avoid demonstrating mere group differences by correlating all groups together. No correction for multiple testing was applied.

There was a significant correlation between CRP and BMI in HC and SZ (HC: r(s) = 0.529, p = 0.04; SZ: r(s) = 0.398, p = 0.01; MDD: r(s) = 0.234, p = 0.18), while there were neither significant correlations between CCL20 and BMI (HC: r(s) = 0.089, p = 0.75; SZ: r(s) = − 0.015, p = 0.93; MDD: r(s) = 0.28, p = 0.10) nor between TRAIL and BMI (HC: r(s) = 0.189, p = 0.50; SZ: r(s) = 0.078, p = 0.63; MDD: r(s) = 0.027, p = 0.88) (analysis were controlled for smoking and sex).

Further, there were no significant correlations between smoking and CRP (HC: r(s) = 0.152, p = 0.59; SZ: r(s) = 0.302, p = 0.06; MDD: r(s) = − 0.089, p = 0.62) nor CCL20 (HC: r(s) = 0.057, p = 0.84; SZ: r(s) = 0.190, p = 0.24; MDD: r(s) = 0.320, p = 0.054) nor TRAIL (HC: r(s) = 0.371, p = 0.17; SZ: r(s) = 0.258, p = 0.11; MDD: r(s) = 0.267, p = 0.12) (analyses were controlled for BMI and sex).

### CRP does not correlate with CCL20 or TRAIL

To investigate if CCL20 and TRAIL were independent of CRP, we used correlational analyses. Neither significant correlations between CRP and CCL20 (HC: r(s) = 0.081, p = 0.78; SZ: r(s) = 0.144, p = 0.38; MDD: r(s) = 0.266, p = 0.13) nor between CRP and TRAIL (HC: r(s) = − 0.086, p = 0.77; SZ: r(s) = − 0.160, p = 0.33; MDD: r(s) = 0.197, p = 0.27) were present (analyses were controlled for BMI, sex and smoking).

Further, we investigated the inter-relationship of CCL20 and TRAIL and observed no significant correlations (HC: r(s) = 0.114, p = 0.69; SZ: r(s) = 0.191, p = 0.24; MDD: r(s) = 0.103, p = 0.56) (analyses were controlled for BMI, sex and smoking).

## Discussion

In the current study, we investigated differences in 75 peripheral inflammatory markers including the acute phase protein CRP between patients with MDD, SZ and HC. After correcting for BMI, sex and smoking as covariates and correcting for multiple testing within the 74 inflammatory markers, significant differences emerged for CCL20 and TRAIL. TRAIL differed in both diagnosis groups (SZ and MDD) from HC. Levels of CCL20 were increased in patients with SZ compared to healthy controls and to patients with MDD, suggesting a potential disease specificity.

Findings of increased CRP in MDD and SZ have been replicated abundantly in the literature, but have also been demonstrated to depend strongly on the sample collection procedures and analysis methods^21,26,27,33^. In the current study CRP was increased in patients with SZ compared to both other groups, but results remained no longer significant after introducing BMI as covariate. This might be due to the fact that BMI has been extensively associated with increased CRP^37^ and points to the challenge of disentangling the metabolic underpinnings of mental disorder and physical comorbidity with metabolic aspects^21^.

Importantly, we demonstrate a robust increase in TRAIL in patients with both MDD and SZ compared to HC, while CCL20 was specifically increased in patients with SZ compared to patients with MDD and HC, indicating a specificity for SZ. Due to their common involvement in inflammatory processes, we further followed up on the relationship of TRAIL and CCL20 with CRP within diagnostic groups. We observed no significant correlations of CRP with TRAIL and CCL20, demonstrating that TRAIL and CCL20 are inflammatory markers independent of CRP. We demonstrated further, as opposed to CRP, that the observed effects in TRAIL and CCL20 are independent of BMI.

TRAIL (also known as TNFSF10) is a cytokine and member of the TNF superfamily. Upon binding to its receptors, TRAIL can activate apoptosis and pro-inflammatory effects and was identified based on its sequence homology to TNF^38^. TRAIL is involved in immune homeostasis and tumor suppression and acts by triggering the extrinsic apoptotic pathway by interacting with death receptors DR4 (TRAILR1) and DR5 (TRAILR2)^39^. TRAIL dependent neurotoxic effects have been described in hypoxic-ischemic brain damage and in HIV encephalopathy^40,41^. Neuronal cell death has been postulated as mechanism in MDD and SZ, however also conflicting evidence exists^42–45^.

In rodent studies, it has been demonstrated that a peripheral increase of TRAIL does not yield detectable levels of TRAIL in the brain in the presence of an intact blood brain barrier (BBB)^46^. However, given the recent evidence that MDD and SZ are associated with a damage of the BBB^47,48^, further studies are warranted on the relationship between peripheral and central levels of TRAIL in the presence of an altered BBB permeability.

Peripheral increases of TRAIL have been suggested as a marker for brain disorders such as neurocognitive impairment and depression based on preliminary evidence^49^. TRAIL-Receptor (R) 4, was demonstrated to be overexpressed in individuals with MDD reporting childhood trauma, suggesting a relationship between depression and cytokine alterations^50^. In a study investigating cocaine withdrawal, TRAIL was positively associated with depression severity and with TNF-alpha levels^39^. In schizophrenia, a study aiming at creating a blood-based laboratory test to predict the presence of SZ in patients vs HC defined an immunoassay panel consisting of 51 immune markers, among them TRAIL R3 that could distinguish patients with SZ from HC^51^. Further, genetic analyses indicate an implication of TRAIL gene expression in schizophrenia^52^ and demonstrated a contribution to an algorithm for classification of schizophrenia versus controls^53^.

It has been demonstrated in rodents that peripheral TRAIL is upregulated under inflammatory conditions and that inhibition of TRAIL-signaling reduces the production of CCL20 and homing of T cells, limiting T_H_2 cytokine release and subsequent inflammation^54^. This emphasizes the role of TRAIL and CCL20 in the link between innate and adaptive immune system, potentially also in mental health and is in line with observations of increased NMDAR antibodies in patients with schizophrenia and major depressive disorder, providing a link to autoimmunity as pathomechanisms in mental illness^32,55^. In our study, we observed no correlation between TRAIL and CCL20, supporting the conclusion, that CCL20 is an inflammatory marker specific for schizophrenia, while TRAIL is a transdiagnostic marker for SZ and MDD, with possibly different underlying pathways or non-linear relationships.

CCL20 is the only known chemokine ligand to the chemokine receptor CCR6 and is expressed by various cell types in both peripheral blood mononuclear cells and central nervous system cells^56,57^. It was shown that in contrast to TRAIL, CCL20 can access the brain under physiological conditions. CCL20 is excessively present in the choroid plexus and subarachnoid space, providing a link between the periphery and brain, with CCL20 possibly easily entering the brain from systemic circulation^58^. In inflammatory conditions, CCL20 is upregulated and under experimental conditions, various cytokines have been found to induce CCL20 expression. The finding that opposing cell subtypes (T_H_17 and T_reg_ cells) express and respond to CCL20 hints at a potential regulatory role between immune activation and suppression^59^. CCL20 is a chemokine involved in chemotaxis and inflammatory response and has been described to be involved in several inflammation-related conditions, e.g. rheumatoid arthritis^59^. Inflammatory mediators can lead to the production of CCL20 in microglia and can alter T cell behavior, which proposes a potential role of CCL20 in neuroinflammation and progressive neuropathological processes in mental disorders^60^. Regarding SZ, one review indicated an increase of a cluster in gene expression analysis containing among others CCL20, that was overexpressed in monocytes from SZ patients compared to HC^61^. Another study demonstrated a reduced increase in gene expression of CCL20 in SZ-derived iPSC-astrocytes upon stimulation with IL-1b^56^. The hyporesponsiveness of SZ derived astrocytes to IL-1b suggests that cellular CCL20 pathways might be differentially affected in peripheral and brain tissue in SZ. In the light of CCL20 exerting potent chemotactic effects on T_reg_ cells, the attraction of T_reg_ cells from periphery to the brain might be dysregulated in SZ^56^ with the result that T_reg_ cells are held back in the periphery and fail to be recruited to the brain. The concept outlined here is supported by the finding of increased peripheral T_reg_ cells in SZ vs HC^62^.

While our findings suggest that elevated peripheral CCL20 may be specific to schizophrenia and not to MDD, it has to be noted that an increased expression of CCL20 coding mRNA in monocytes of patients with bipolar disorder has been described^63^. However, to our knowledge an increase of circulating CCL20 has not been reported in patients with mood disorders.

These findings demonstrate the importance of considerations beyond pure single group comparisons and support a complex involvement of the immune system, especially in the delineation of schizophrenia pathology from other disorders such as MDD. Further research is needed on inflammation in mental disorders, with a broader focus beyond the investigation of single, canonical markers.

The limitations of the current study pertain mainly to the sample size, which implies that only large effect sizes will reach significance. Therefore, our study has limited power to detect medium or small effects: Due to the correction for multiple testing, smaller effects might not be seen, although they might be functionally important. Thus, larger studies are clearly needed to confirm the present results and to provide a comprehensive assessment of disorder-specific and transdiagnostic inflammatory markers. Further, due to technical reasons, several other inflammatory markers, among them TNFα and IFN-γ, could not be analyzed.

Another challenge pertains the handling of covariates: here, we determined covariates mainly based on demographic group differences, but an approach employing further covariates based on their hypothetic role in inflammation could be considered with a larger sample size. However, accounting for heterogeneity and forming hypothesis-based subgroups might be a promising approach in a study with a larger sample size.

Additionally, the majority of our patients were medicated, with antipsychotic and antidepressant medication having been demonstrated to influence inflammatory levels. Although we did not find significant correlations of inflammatory markers and medication dose within most groups, we cannot exclude that medication contributed to group differences. This should be further investigated in larger studies with specific hypotheses based on in-vitro findings^64^. To exclude an influence of psychotropic medication on the observed results in this study it would be necessary to conduct studies with unmedicated patients. Further, antipsychotic and antidepressant medication were demonstrated to lead to weight gain, which might underlie the observed differences in BMI^65^.

To conclude, we demonstrate that CCL20 is a novel and CRP-independent marker with the potential of being diagnosis-specific for schizophrenia, and TRAIL is a transdiagnostic marker for depression and schizophrenia, while further studies on the influence of psychotropic medication are warranted to confirm this finding. Upregulation of CCL20 is a highly interesting candidate mechanism potentially specific for schizophrenia and further knowledge is needed on the implications of altered peripheral levels of CCL20 in patients with SZ. Identifying novel biomarkers in mental disease by employing a transdiagnostic approach, bears the potential for further research towards novel treatment strategies for difficult-to-treat symptoms by possibly modifying inflammation-related processes.

## Methods

### Participants

42 patients meeting the DSM-IV (American Psychiatric Association, 2000) criteria for schizophrenia, 37 patients meeting the criteria for MDD and 17 healthy control participants were included in the analysis of the present study after exclusion of patients whose samples failed quality control (see inflammatory markers below). Diagnoses were confirmed by conducting the Mini-International Neuropsychiatric Interview (Lecrubier et al., 1999). Patients were recruited from outpatient and inpatient units of the Psychiatric Hospital of the University of Zurich and affiliated institutions. All patients were clinically stable and under a stable dose of medication for at least two weeks prior to testing. Inpatients were at the end of their hospitalization and engaged in a multimodal therapy program and activities outside the hospital. Please note that the average duration of hospitalization for patients with schizophrenia and depression in Swiss psychiatric hospitals is longer than in most other countries, so the majority of inpatients would have been treated as outpatients in other health care systems. Healthy controls were recruited from the community via advertisement. The inclusion age was between 18 and 65 years. More patients than controls were recruited to have adequate power for the correlational analyses (see below). All participants gave written informed consent and the project was approved by the Ethics Committee of the Canton of Zurich.

We excluded patients with any other than the above-mentioned DSM-IV Axis I disorders, lorazepam medication higher than 1 mg per day and acute psychotic symptoms. Participants with any alcohol use disorder based on lifetime criteria and participants with current abuse or dependency of cannabis were excluded. Healthy controls were excluded if any psychiatric diagnosis was present in the structured Mini-International Neuropsychiatric Interview.

In all groups, participants were excluded, if they had any autoimmune or chronic inflammatory disorder or if they took any pain-medication or anti-inflammatory drugs at least one week prior to testing. Furthermore, participants were not included in the study if they had a history of any acute infection two weeks prior to testing. This information was assessed using questionnaires and if available by review of medical charts.

We have previously published findings on task-based explorative behavior in a population of patients with SZ partially overlapping with the current sample^66–69^, which used some of the same inflammation measures in the SZ population^70^. In the current publication, we specifically compare additionally inflammatory markers of MDD patients with SZ patients to assess specificity to individual disease.

### Ethics approval and consent to participate

All participants gave written informed consent and the project was approved by the Ethics Committee of the Canton of Zurich, Switzerland.

All methods were carried out in accordance with relevant guidelines and regulations.

### Inflammatory marker assessment

#### Blood draw

Blood was drawn between 8 and 10 am on the same day prior to testing. Study participants were instructed to fast for at least 8 h prior to the blood draw and this was verified by a questionnaire on the day of testing. To obtain plasma (Olink panel), blood was drawn into ethylenediaminetetraacetic acid (EDTA) tubes (Sarstedt, Switzerland), centrifuged for 15 min at 1500 g and plasma was frozen at − 80 °C. High sensitivity (hs) CRP was measured in serum: blood was collected into a silica and gel containing tube (BD Vacutainer) and processed as described below.

#### High-sensitive C-reactive protein (CRP)

CRP was measured in patient serum samples by immunoturbidimetry on Abbott Architect c8000.

#### Inflammatory markers

Proteins were measured using the Olink® Inflammation panel (Olink Proteomics AB, Uppsala, Sweden) according to the manufacturer's instructions (https://www.olink.com/products/inflammation). The Proximity Extension Assay (PEA) technology used for the Olink protocol has been well described (Assarsson et al., 2014), and enables 92 analytes to be analyzed simultaneously, using 1 µL of each sample. In brief, pairs of oligonucleotide-labeled antibody probes bind to their targeted protein, and if the two probes are brought in close proximity the oligonucleotides will hybridize in a pair-wise manner. The addition of a DNA polymerase leads to a proximity-dependent DNA polymerization event, generating a unique PCR target sequence. The resulting DNA sequence is subsequently detected and quantified using a microfluidic real-time PCR instrument (Biomark HD, Fluidigm). Data is then quality-controlled and normalized using an internal extension control and an inter-plate control, to adjust for intra- and inter-run variation. The final assay read-out is presented in Normalized Protein eXpression (NPX) values, which is an arbitrary unit on a log2-scale where a high value corresponds to a higher protein expression. All assay validation data (detection limits, intra- and inter-assay precision data, etc.) are available on the manufacturer's website (https://www.olink.com/content/uploads/2019/04/Olink-Inflammation-Validation-Data-v3.0.pdf). Values below limit of detection (LOD) were replaced with the value for LOD for the specific assay (the proportion of replacement was on average 6%, depending on the protein). 12 participants (2 HC, 3 SZ, 7 MDD) were excluded from the larger dataset prior to all analyses because their samples failed quality control. Four internal controls were added to each sample to monitor the quality of assay performance, as well as the quality of individual samples. The quality control (QC) was performed in two steps: First, each sample plate was evaluated on the standard deviation of the internal controls, which should be below 0.2 NPX. Only data from sample plate that passed this quality control were reported. Second, the quality of each sample was assessed by evaluating the deviation from the median value of the controls for each individual sample. Samples that deviated less than 0.3 NPX from the median passed the quality control. 74 of total 92 proteins could be detected in > 80% of samples (no analysis of ARTN, BDNF, IFN-gamma, IL-1 alpha, IL-13, IL2, IL-20, IL-20RA, IL-22 RA1, IL-24, IL-2RB, IL-33, IL-4, IL-5, LIF, NRTN, TNF, TSLP, see Supplemental Table 1 for abbreviations). Inter-assay coefficient of variation (CV) distribution was 60 proteins with below 10%, 30 proteins for 10–20% and 1 protein for 20–30%. Intra-assay CV distribution was 41 proteins below 5%, 41 proteins from 5 to 10%, 7 proteins from 10 to 15% and 2 proteins above 15%.

### Statistical analysis

Statistical analysis was performed using SPSS version 25 (IBM Corp., SPSS Inc., Chicago IL, USA). The Kolmogorov Smirnov-test was used to assess normality of distribution and CRP was log_10_-transformed to achieve normal distribution. Inflammatory markers of the Olink panel are presented as normalized values (NPX). Statistical tests report two-sided p-values and the level of significance was set at p < 0.05. The estimate of variance used is one standard deviation (SD).

#### Sample characterization

Group differences between demographic variables were calculated using analysis of variance (ANOVA) with post-hoc testing using the Benjamini–Hochberg procedure to reduce the false discovery rate (FDR)^71^ for comparison of all three diagnostic groups. For comparison of the two patient groups, t-test and Mann Whitney U test were used as appropriate. For the analysis of sex, Pearson’s Chi Square was used. Based on significant differences between groups, BMI was further used as covariate in all inflammatory marker-related analyses. Despite the absence of significant differences between groups, smoking and sex were also used as covariates in all analyses based on the literature^36^.

#### Group analyses of inflammatory markers and CRP

For group analyses of inflammatory markers of the OLINK panel and CRP, a one-way analysis of variance (ANOVA) was used to investigate the differences of the inflammatory markers between the three diagnostic groups. CRP was analyzed independently due to its characteristic as a marker for overall inflammatory activity, while the markers of the OLINK panel were analyzed to investigate specific inflammatory markers. To correct for multiple comparisons, overall significant values of the ANOVA of inflammatory markers were corrected using the Benjamini–Hochberg procedure^71^. Post-hoc comparisons also using the Benjamini–Hochberg procedure^71^ were applied only to inflammatory marker parameters that remained significant after overall FDR-correction of main effects. Analysis of covariance (ANCOVA) was conducted for CRP and inflammatory markers with BMI, sex and smoking as covariates, correction for multiple comparison was conducted using the Benjamini–Hochberg procedure^71^. Effect sizes were calculated using Cohen’s d.

#### Correlational analyses

For correlational analysis between inflammatory markers with significant differences in group analysis (namely TRAIL and CCL20) and CRP with potential confounding factors, we selected BMI and smoking (pack years of current smokers as indicator of long-term smoking) due to their role in inflammatory measurements based on the literature^36^. We computed non-parametric partial correlations (correlation coefficient r(s) (Spearman’s rho) due to non-normal distribution of variables) separately per group in order to avoid depicting mere group differences. Due to the exploratory nature of this analysis, no correction for multiple testing was applied. The level of significance was set at p < 0.05. We controlled for sex and BMI (in the correlation with smoking) and for sex and smoking (in the correlation with BMI) as covariates.

The two inflammatory markers for which significant differences in group analysis were observed (CCL20 and TRAIL) were further used to investigate their inter-relationship and their relationship with CRP in the three groups separately using correlational analysis. The same strategy as describe above, with BMI (based on significant group differences) and sex and smoking (based on the literature^36^) as covariates was used.

## Supplementary Information

Supplementary Information.
