# Supplementary material for: Common and disorder-specific upregulation of the inflammatory markers TRAIL and CCL20 in depression and schizophrenia
Source: Sci Rep. 2021 Sep 28;11:19204. doi: 10.1038/s41598-021-98769-0 (PMC8479067; doi:10.1038/s41598-021-98769-0)
Supplement: Supplementary file 1 — Supplementary Information. [file 41598_2021_98769_MOESM1_ESM.docx]

**Common and disorder-specific upregulation of the inflammatory markers TRAIL and CCL20 in depression and schizophrenia**

Federica Klaus^a,b^ *, Karoline Guetter^a^, Rebecca Schlegel^a^, Tobias R. Spiller^c^, Erich Seifritz^a^, Flurin Cathomas^a,d 1^ , Stefan Kaiser^e 1^

^a^ Department of Psychiatry, Psychotherapy and Psychosomatics, Psychiatric Hospital, University of Zurich, 8032 Zurich, Switzerland

^b^ Department of Psychiatry, University of California San Diego, San Diego, USA

^c^ Department of Consultation-Liaison Psychiatry and Psychosomatic Medicine, University Hospital Zurich, University of Zurich, 8091 Zurich, Switzerland

^d^ Fishberg Department of Neuroscience, Friedman Brain Institute, Icahn School of

Medicine at Mount Sinai, New York, USA

^e^ Division of Adult Psychiatry, Department of Psychiatry, Geneva University Hospitals, Chemin du Petit-Bel-Air, 1225 Chêne-Bourg, Switzerland

^*^ Corresponding author

^1^  These authors contributed equally

**Supplementary Table S1: List of abbreviation of inflammatory biomarkers**

| **Abbreviation** | **Legend** |
| --- | --- |
| ADA | Adenosine Deaminase |
| AXIN1 | Axin-1 |
| Beta-NGF | Beta-nerve growth factor |
| CASP-8 | Caspase-8 |
| CCL | C-C motif chemokine |
| CD | Cluster of differentiation |
| CDCP1 | CUB domain-containing protein 1 |
| CRP | C-reactive protein |
| CSF-1 | Colony-stimulating factor 1 |
| CST5 | Cystatin-D |
| CXCL | C-X-C motif chemokine |
| DNER5 | Delta and notch-like epidermal growth factor-related receptor |
| EN-RAGE | Protein S100-A12 |
| Flt3L | Fms-related tyrosine kinase 3 ligand |
| FGF | Fibroblast growth factor |
| GDNF | Glial cell line-derived neurotrophic factor |
| HGF | Hepatocyte growth factor |
| IL | Interleukin |
| IL-R | Interleukin receptor |
| LAPTGF | Latency-associated peptide transforming growth factor beta-1 |
| Lif-R | Leukemia inhibitory factor receptor |
| MMP | Matrix metalloproteinase |
| NT-3 | Neurotrophin-3 |
| OPG | Osteoprotegerin |
| OSM | Oncostatin-M |
| PD-L1 | Programmed cell death 1 ligand 1 |
| SCF | Stem cell factor |
| TNFB | Tumor necrosis factor beta |
| TNFRSF9 | Tumor necrosis factor receptor superfamily member 9 |
| TNFSF14 | Tumor necrosis factor ligand superfamily member 14 |
| TRANCE | Tumor necrosis factor-related activation-induced cytokine |
| TWEAK | Tumor necrosis factor (ligand) superfamily, member 12 |
| TRAIL | Tumor necrosis factor related apoptosis-inducing ligand |
| SLAMF1 | Signaling lymphocytic activation molecule family member 1 |
| SIRT2 | SIR2-like protein 2 |
| ST1A1 | Sulfotransferase 1A1 |
| STAMPB | STAM-binding protein |
| uPA | Urokinase-type plasminogen activator |
| VEGFA | Vascular endothelial growth factor |

**Supplementary Table S2: Group comparison of biomarkers**

| **Biomarker** | **HC** | | | **SZ** | | | **MDD** | | | **Main effect** | **Critical value^1^** |
| --- | --- | --- | --- | --- | --- | --- | --- | --- | --- | --- | --- |
|  | **n^2^** | **Mean** | **SD** | **n^2^** | **mean** | **SD** | **n^2^** | **mean** | **SD** | **p** | **p** |
| **TRAIL** | 17 | 7.939 | 0.439 | 42 | 8.310 | 0.243 | 37 | 8.182 | 0.234 | **< 0.001** | 0.001 |
| **CCL20** | 17 | 4.547 | 0.574 | 42 | 5.551 | 1.263 | 37 | 4.760 | 0.617 | **< 0.001** | 0.001 |
| DNER | 17 | 7.942 | 0.238 | 42 | 7.985 | 0.290 | 37 | 7.791 | 0.198 | 0.003 | 0.002 |
| HGF | 17 | 7.698 | 0.340 | 42 | 7.821 | 0.358 | 37 | 7.535 | 0.382 | 0.003 | 0.003 |
| IL18 | 17 | 7.487 | 0.470 | 17 | 7.487 | 0.470 | 37 | 7.192 | 0.481 | 0.005 | 0.003 |
| FGF21 | 17 | 4.559 | 1.353 | 42 | 5.342 | 1.203 | 37 | 5.822 | 1.419 | 0.006 | 0.004 |
| OSM | 17 | 2.314 | 0.695 | 42 | 2.466 | 0.975 | 37 | 1.836 | 0.810 | 0.006 | 0.005 |
| MCP3 | 16 | 0.822 | 0.366 | 38 | 1.145 | 0.526 | 35 | 0.829 | 0.408 | 0.007 | 0.005 |
| CCL11 | 17 | 7.161 | 0.383 | 42 | 7.520 | 0.478 | 37 | 7.312 | 0.363 | 0.008 | 0.006 |
| IL6 | 17 | 2.467 | 0.509 | 42 | 3.008 | 0.907 | 37 | 2.633 | 0.668 | 0.022 | 0.007 |
| IL10RB | 17 | 6.377 | 0.249 | 42 | 6.427 | 0.265 | 37 | 6.258 | 0.308 | 0.030 | 0.007 |
| OPG | 17 | 10.312 | 0.379 | 42 | 10.154 | 0.288 | 37 | 10.058 | 0.382 | 0.044 | 0.008 |
| MCP1 | 17 | 9.541 | 0.367 | 42 | 9.783 | 0.427 | 37 | 9.593 | 0.390 | 0.046 | 0.009 |
| TRANCE | 17 | 4.055 | 0.741 | 42 | 4.470 | 0.674 | 37 | 4.239 | 0.506 | 0.054 | 0.009 |
| SCF | 17 | 9.651 | 0.394 | 42 | 9.398 | 0.421 | 37 | 9.533 | 0.375 | 0.072 | 0.010 |
| CD6 | 17 | 4.451 | 0.453 | 42 | 4.194 | 0.495 | 37 | 4.144 | 0.463 | 0.085 | 0.011 |
| MCP2 | 17 | 7.887 | 0.590 | 42 | 7.618 | 0.743 | 37 | 7.479 | 0.492 | 0.092 | 0.011 |
| TWEAK | 17 | 9.328 | 0.417 | 42 | 9.196 | 0.427 | 37 | 9.077 | 0.360 | 0.096 | 0.012 |
| CSF1 | 17 | 7.780 | 0.248 | 42 | 7.764 | 0.227 | 37 | 7.662 | 0.238 | 0.096 | 0.013 |
| LAPTGFβ1 | 17 | 7.568 | 0.720 | 42 | 7.226 | 0.602 | 37 | 7.219 | 0.515 | 0.099 | 0.014 |
| ADA | 17 | 3.962 | 0.612 | 42 | 3.748 | 0.619 | 37 | 3.604 | 0.476 | 0.099 | 0.014 |
| CCL28 | 17 | 1.104 | 0.434 | 42 | 0.886 | 0.469 | 37 | 1.061 | 0.390 | 0.108 | 0.015 |
| CDCP1 | 17 | 2.205 | 0.514 | 42 | 2.451 | 0.532 | 37 | 2.210 | 0.617 | 0.118 | 0.016 |
| Beta NGF | 17 | 1.543 | 0.238 | 42 | 1.439 | 0.166 | 37 | 1.517 | 0.238 | 0.131 | 0.016 |
| CXCL11 | 17 | 7.851 | 1.029 | 42 | 7.234 | 1.152 | 37 | 7.382 | 1.014 | 0.143 | 0.017 |
| CD5 | 17 | 4.772 | 0.266 | 42 | 4.747 | 0.417 | 37 | 4.592 | 0.421 | 0.152 | 0.018 |
| IL17C | 17 | 1.375 | 0.422 | 42 | 1.609 | 0.558 | 37 | 1.432 | 0.482 | 0.170 | 0.018 |
| CD40 | 17 | 10.141 | 0.675 | 42 | 9.909 | 0.620 | 37 | 9.812 | 0.556 | 0.186 | 0.019 |
| CD244 | 17 | 6.057 | 0.629 | 42 | 5.838 | 0.564 | 37 | 5.749 | 0.544 | 0.187 | 0.020 |
| FGF23 | 17 | 1.848 | 0.507 | 42 | 1.951 | 0.361 | 37 | 1.791 | 0.374 | 0.197 | 0.020 |
| VEGFA | 17 | 9.307 | 0.444 | 42 | 9.330 | 0.501 | 37 | 9.158 | 0.370 | 0.209 | 0.021 |
| CASP8 | 17 | 1.951 | 1.262 | 42 | 1.548 | 0.866 | 37 | 1.517 | 0.813 | 0.246 | 0.022 |
| CXCL10 | 17 | 7.750 | 1.038 | 42 | 7.416 | 0.745 | 37 | 7.671 | 0.829 | 0.256 | 0.022 |
| Flt3L | 17 | 8.477 | 0.516 | 42 | 8.539 | 0.535 | 37 | 8.358 | 0.429 | 0.265 | 0.023 |
| CX3CL1 | 17 | 4.922 | 0.354 | 42 | 4.811 | 0.354 | 37 | 4.753 | 0.352 | 0.266 | 0.024 |
| TNFRSF9 | 17 | 5.724 | 0.405 | 42 | 5.873 | 0.404 | 37 | 5.744 | 0.403 | 0.267 | 0.024 |
| CXCL6 | 17 | 8.193 | 1.268 | 42 | 7.853 | 1.064 | 37 | 7.703 | 0.853 | 0.272 | 0.025 |
| IL7 | 17 | 3.915 | 0.932 | 42 | 3.620 | 0.874 | 37 | 3.519 | 0.749 | 0.275 | 0.026 |
| CCL25 | 17 | 5.740 | 0.650 | 42 | 5.962 | 0.555 | 37 | 5.790 | 0.703 | 0.345 | 0.026 |
| IL18R1 | 17 | 6.658 | 0.457 | 42 | 6.707 | 0.329 | 37 | 6.571 | 0.502 | 0.370 | 0.027 |
| TNFSF14 | 17 | 4.532 | 0.911 | 42 | 4.428 | 0.702 | 37 | 4.244 | 0.793 | 0.382 | 0.028 |
| FGF5 | 16 | 1.003 | 0.204 | 41 | 0.930 | 0.200 | 37 | 0.981 | 0.218 | 0.389 | 0.028 |
| CCL3 | 17 | 4.204 | 0.497 | 42 | 4.350 | 0.560 | 37 | 4.413 | 0.653 | 0.481 | 0.029 |
| FGF19 | 17 | 7.955 | 0.928 | 42 | 7.674 | 0.832 | 37 | 7.701 | 0.816 | 0.491 | 0.030 |
| ST1A1 | 17 | 2.960 | 1.347 | 42 | 2.679 | 1.004 | 37 | 2.912 | 0.853 | 0.493 | 0.030 |
| SLAMF1 | 17 | 1.271 | 0.461 | 42 | 1.148 | 0.372 | 37 | 1.150 | 0.367 | 0.500 | 0.031 |
| GDNF | 17 | 1.384 | 0.406 | 42 | 1.334 | 0.430 | 37 | 1.260 | 0.317 | 0.500 | 0.032 |
| CXCL9 | 17 | 6.819 | 1.053 | 42 | 6.630 | 0.543 | 37 | 6.807 | 0.789 | 0.504 | 0.032 |
| CCL23 | 17 | 9.635 | 0.393 | 42 | 9.492 | 0.476 | 37 | 9.583 | 0.538 | 0.533 | 0.033 |
| IL17A | 14 | 0.206 | 0.354 | 33 | 0.392 | 0.704 | 32 | 0.336 | 0.418 | 0.569 | 0.034 |
| IL8 | 17 | 5.177 | 0.562 | 42 | 5.274 | 0.575 | 37 | 5.152 | 0.492 | 0.586 | 0.034 |
| ENRAGE | 17 | 1.637 | 0.870 | 42 | 1.757 | 0.753 | 37 | 1.596 | 0.812 | 0.656 | 0.035 |
| MMP1 | 17 | 11.979 | 1.461 | 42 | 11.681 | 1.165 | 37 | 11.763 | 0.945 | 0.666 | 0.036 |
| CCL4 | 17 | 6.122 | 0.480 | 42 | 6.175 | 0.646 | 37 | 6.280 | 0.814 | 0.686 | 0.036 |
| CXCL5 | 17 | 11.278 | 1.470 | 42 | 10.964 | 1.162 | 37 | 11.092 | 1.365 | 0.697 | 0.037 |
| K4EBP1 | 17 | 8.693 | 1.495 | 42 | 8.551 | 1.080 | 37 | 8.417 | 1.047 | 0.703 | 0.038 |
| uPA | 17 | 10.243 | 0.238 | 42 | 10.288 | 0.299 | 37 | 10.216 | 0.533 | 0.722 | 0.039 |
| CST5 | 17 | 5.820 | 0.490 | 42 | 5.704 | 0.574 | 37 | 5.774 | 0.548 | 0.726 | 0.039 |
| CCL19 | 17 | 8.629 | 1.090 | 42 | 8.577 | 0.617 | 37 | 8.460 | 0.969 | 0.748 | 0.040 |
| IL10 | 17 | 2.731 | 1.281 | 42 | 2.671 | 0.521 | 37 | 2.592 | 0.493 | 0.773 | 0.041 |
| MMP10 | 17 | 5.337 | 0.455 | 42 | 5.465 | 0.682 | 37 | 5.418 | 0.635 | 0.779 | 0.041 |
| CXCL1 | 17 | 8.769 | 1.016 | 42 | 8.591 | 0.925 | 37 | 8.680 | 0.850 | 0.780 | 0.042 |
| TGFalpha | 17 | 2.342 | 0.254 | 42 | 2.376 | 0.255 | 37 | 2.393 | 0.257 | 0.798 | 0.043 |
| STAMPB | 17 | 5.591 | 1.801 | 42 | 5.345 | 1.253 | 37 | 5.376 | 1.144 | 0.804 | 0.043 |
| SIRT2 | 17 | 4.370 | 2.113 | 42 | 4.109 | 1.475 | 37 | 4.087 | 1.475 | 0.817 | 0.044 |
| IL10RA | 17 | 1.439 | 0.978 | 42 | 1.356 | 1.290 | 35 | 1.242 | 0.926 | 0.817 | 0.045 |
| LIFR | 17 | 2.299 | 0.247 | 42 | 2.293 | 0.232 | 37 | 2.267 | 0.285 | 0.879 | 0.045 |
| IL15RA | 17 | 0.106 | 0.185 | 42 | 0.113 | 0.204 | 36 | 0.092 | 0.165 | 0.884 | 0.046 |
| IL12B | 17 | 4.096 | 0.657 | 42 | 4.000 | 0.779 | 37 | 4.037 | 0.571 | 0.885 | 0.047 |
| NT3 | 17 | 1.820 | 0.337 | 42 | 1.811 | 0.389 | 37 | 1.777 | 0.382 | 0.897 | 0.047 |
| MCP4 | 17 | 3.491 | 0.742 | 42 | 3.402 | 0.765 | 37 | 3.406 | 0.593 | 0.897 | 0.048 |
| TNFB | 17 | 3.569 | 0.312 | 42 | 3.580 | 0.447 | 37 | 3.539 | 0.395 | 0.902 | 0.049 |
| PDL1 | 17 | 3.576 | 0.359 | 42 | 3.519 | 0.338 | 37 | 3.549 | 0.606 | 0.906 | 0.049 |
| AXIN1 | 17 | 5.129 | 2.066 | 42 | 5.093 | 1.395 | 37 | 5.104 | 1.160 | 0.996 | 0.050 |

Units are normalized protein expression (NPX) (an arbitrary unit on a log2-scale where a high value corresponds to a higher protein expression). Mean and standard deviation (SD) are depicted.

Abbreviations: HC: Healthy control participants, MDD: major depression, SZ: schizophrenia. For abbreviations of biomarkers please refer to table S1.

^1^ Group comparison was conducted using ANOVA, then the Benjamini-Hochberg procedure to reduce FDR was applied (37). Biomarker with significant overall group differences (main effect of group) after FDR correction are in bold, variables are sorted in descending order of significance after FDR correction.

^2^ sample size varies due to technical aspects (e.g. sample did not meet quality control).
